# The Microstructure Evolution and Mechanical Properties of Rotary Friction Welded Duplex Stainless Steel Pipe

**DOI:** 10.3390/ma16093569

**Published:** 2023-05-06

**Authors:** Shuxin Zhang, Faqin Xie, Xiangqing Wu, Jinheng Luo, Weiwei Li, Xi Yan

**Affiliations:** 1 School of Civil Aviation, Northwestern Polytechnical University, Xi’an 710072, China; zhangshuxin003@cnpc.com.cn (S.Z.);; 2 Tubular Goods Research Institute, China National Petroleum Corporation & State Key Laboratory for Performance and Structure Safety of Petroleum Tubular Goods and Equipment Materials, Xi’an 710065, China; 3 Shaanxi Society for Environmental Sciences, Xi’an 710065, China

**Keywords:** duplex stainless steel, rotary friction weld, solid-state weld, detrimental phase, sigma phases, EBSD

## Abstract

The use of duplex stainless steel (DSS) in various fields is promising due to its excellent anti-corrosion properties, but traditional welding can lead to the formation of unfavorable phases that deteriorate its quality. This study aimed to use the rotary friction weld (RFW) technique to prevent the formation of harmful phases in the welding of an S32205 alloy pipe. The welding parameters used included a rotating speed of 20 m/s, a friction pressure of 10 MPa, a friction time of 30 s, and a forging pressure of 30 MPa. The microstructure and mechanical properties of the resulting RFWed joint were investigated. The results revealed that the weld zone exhibited a microstructure consisting of ferrite and austenite phases, with no deleterious phase detected. The ferrite content was measured to be 53.3%, 54.5%, and 68.7% in the base metal, thermomechanical affected zone (TMAZ), and weld, respectively, owing to the rapid cooling rate in the RFW process, which prevented any harmful phase formation in the weld zone. Furthermore, the RFW process successfully produced an ultrafine grain with a ferrite/austenite grain size of 0.40 μm and 0.41 μm, respectively. The weld zone and TMAZ contained more low-angle grain boundaries (LAGBs) compared to the base metal, which was attributed to the dynamic recovery (DRV) within a grain. The high heating and cooling rates and short welding time of the RFW process did not allow sufficient time for the dynamic recrystallization of the microstructure in the weld zone. However, a slight increase in the ferrite content in the weld zone resulted in grain refinement and an increase in the dislocation density, resulting in a slight increase in the 358 HV0.2 hardness and 823 MPa tensile strength of the weld zone. This study offers a novel approach for obtaining ultrafine grain duplex stainless steel pipes with exceptional mechanical properties through the application of RFW.

## 1. Introduction

Ferrite/austenite duplex stainless steel (DSS) possesses excellent toughness, weldability, good corrosion resistance, and high strength, making it a promising material for numerous key energy fields, including marine engineering, petrochemicals, and nuclear power [1]. However, the welding process, which is typically employed to join components in DSS applications, may lead to the precipitation of unfavorable phases, such as Cr-rich carbides and sigma phases [2], or an unbalanced proportion of ferrite and austenite in the heat-affected zone (HAZ), resulting in local pitting corrosion, preferential corrosion, and mechanical property deterioration [3,4,5]. Andrea Putz [6] adopted a stationary arc to investigate the thermal treatment of multi-pass welding and found that the unfavorable precipitation of the secondary phase was observed at 575–1100 °C in both samples. Consequently, the minimization of undesirable deleterious phases or the balancing of phase proportions in DSS welding is a fundamental issue in this area.

Various welding techniques have been used to weld DSS, including shielded metal arc welding (SMAW), gas tungsten arc welding (GTAW) [7,8,9], flux cored arc welding (FCAW), gas metal arc welding (GMAW) [10], submerged-arc welding (SAW) [11,12,13], electron beam welding (EBW) [14,15], laser beam welding (LBW), plasma arc welding (PAW) [16,17,18], and friction stir welding (FSW). However, each method has distinct advantages and disadvantages associated with the solidification of the materials.

For instance, low-energy-density (0.5~50 kW/cm^2^) arc welding techniques including SMAW, GTAW, FCAW, GMAW, and SAW have the advantages of a low heat input and fast welding speed but may result in weld porosity, slag inclusion, segregation, and solidification cracks. In general, these welding methods require the consideration of factors such as welding materials, shielding gases, the mutual influence of multiple passes, heat input, and cyclic heating and cooling. These factors can lead to the formation of secondary precipitated phases, which further affect the corrosion resistance and mechanical properties of the material. Li et al. [19] investigated the GTAW- and GTAW + SMAW-welded DSS joints, finding that the corrosion resistance of the root weld was poor due to the high ferrite content.

On the other hand, high-energy-density (50~5 × 10^5^ kW/cm^2^) welding methods including EBW, LBW, and PAW have the benefits of a low heat input and high cooling speed but may lead to an excessive ferrite structure in the weld and the precipitation of harmful secondary phases that can deteriorate the toughness and localized corrosion resistance of DSS. Zhang et al. [20] investigated the corrosion resistance property of EBW and post-weld heat-treatment joints, finding that a large amount of Cr_2_N formed at the ferrite grain boundaries and deteriorated the corrosion property; after the heat treatment at various temperature, the Cr_2_N amount decreased and a little second secondary austenite formed, so the corrosion property improved.

Rotary friction welding (RFW) [21,22,23], is a solid-state welding technique that can effectively avoid unfavorable phase precipitation and offers additional benefits. It does not require welding wire, and the metal is extruded by friction to form a defect-free weld joint. Furthermore, the welding cooling speed is fast, and it is not easy to produce precipitated phases that may affect the weld’s quality.

Therefore, in this study, the RFW technique was employed to suppress the formation of deleterious phases in the weld of a S32205 alloy pipe. The microstructure and mechanical properties of the RFWed joint were investigated.

## 2. Material and Methods

### 2.1. Materials

**Figure 1:**
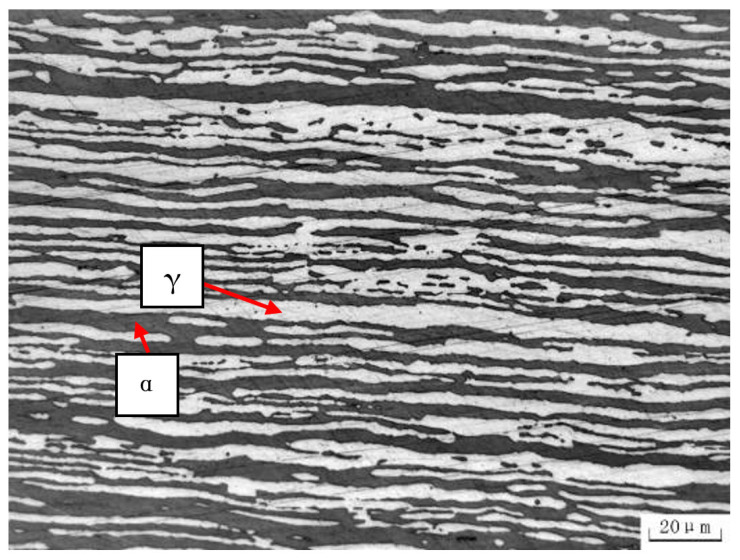
The microstructure of the base metal along the longitudinal direction.

The experimental specimen selected for the study is a two-part longitudinal electric-resistance welded pipe made of S32205 duplex stainless steel, with a diameter of 38.1 mm and a thickness of 3.2 mm. The nominal chemical composition of the material is presented in Table 1. The microstructure of the base material was examined using an Olympus OLS4100 optical microscope (OM, Olympus, Tokyo, Japan), revealing a typical α + γ duplex-phases microstructure, as depicted in Figure 1. The bright phase corresponds to austenite (γ), while the dark gray phase corresponds to ferrite (α). The proportion of ferrite in the microstructure varies across different regions and is approximately 53%.

### 2.2. Determination of Processing Parameters

**Figure 2:**
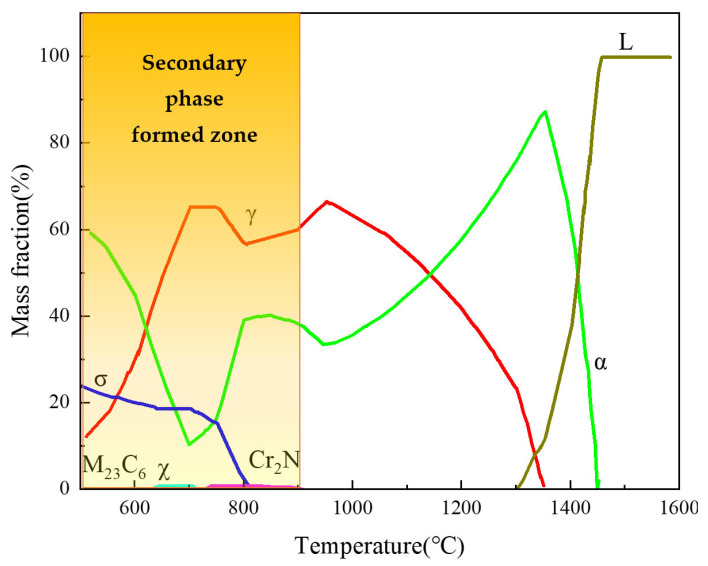
Equilibrium phase diagram of the welding material calculated by FactSage software.

In order to achieve welds that are free of intermetallic precipitation, it is important to understand the equilibrium phase behavior of the base material at different temperatures. The equilibrium phase diagram for duplex stainless steel 2205, as determined using FactSage software and presented in Figure 2, indicates that the formation of a sigma phase should be avoided during welding at low temperatures. Due to the fast-cooling rate of solid-state welding, it is expected to obtain sound joints without secondary phases. In addition, welding parameters were determined based on finite element analysis and in reference to other alloy solid-state welding test results.

**Figure 3:**
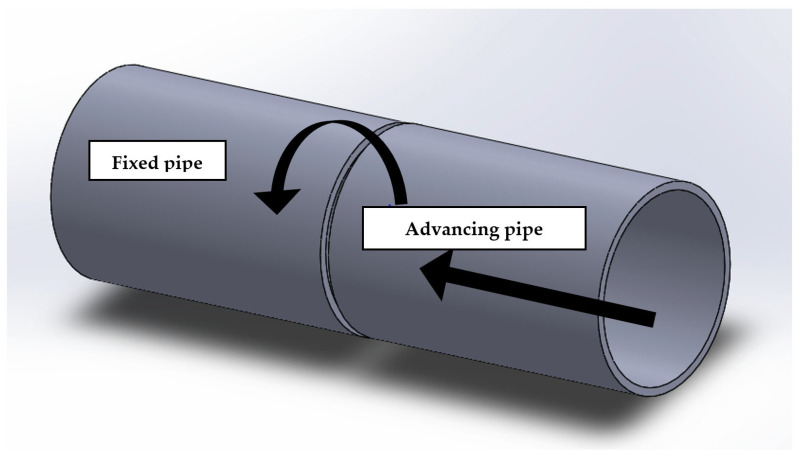
Diagram of rotary friction welding.

A specific welding process was chosen for this study, in which one part of the pipe is held stationary, while the other part is rotated at a speed of 20 m/s, subjected to a friction pressure of 10 MPa and a friction time of 30 s, and followed by a forging pressure of 30 MPa. A schematic of the rotary friction welding process is depicted in Figure 3.

### 2.3. Characterization Method

This study primarily aims to characterize the microstructure and mechanical properties of rotary friction welded joints. The characterization of the microstructure evolution of the welded joint was carried out using various techniques including optical microscopy, energy spectroscopy (EDS), electron backscatter diffraction (EBSD), and transmission electron microscopy (TEM). In addition, the mechanical properties of the welded joint were characterized using tensile properties and hardness tests.

The specimen for optical microscopy examination was wire-cut, then successively ground from 180, 400, 800, 1000, and 1500 to 2000 grit, and polished using diamond pastes of 2.5 μm. The polished metallographic specimen was etched at 90–95 °C for 3 min using a solution of 30 g K_3_Fe(CN)_6_, 30 g KOH, and 100 mL H_2_O. The microstructure was examined using an Olympus LEXT OLS 4100 laser scanning confocal microscope. The EBSD samples were first mechanically polished and then electrolytically polished with a solution containing 57 mL glycerin, 50 mL phosphoric acid, and 7 mL deionized water at 90 °C, at 30 V for 10 min. X-ray diffraction (XRD, Rigaku Smart Lab, Tokyo, Janpan) was utilized to analyze the phase of the weld joint, with six regions of interest tested from the base metal to the other side of the base metal. The EBSD data were analyzed with Channel 5, and the EBSD was examined by TESCAN MIRA3. The TEM samples were first grounded to 0.1 mm and then electrolytic-jet-thinned with a solution containing 10% perchloric acid and 90% ethanol, at 0 °C under 12 V. The TEM experiment was carried out using JEOL-2100F, and images were analyzed using digital micrograph 3 software (Gatan).

The tensile test was conducted at room temperature using a universal testing machine (SANS, Shenzhen, China, UTM 5305) with a crosshead speed of 1 mm/min, in accordance with ASTM A370. Two more samples were tested to prove the reproducibility of the result. The HV0.2 Vickers hardness was measured across the weld line using a hardness tester (KB Prüftechnik, Hochdorf-Assenheim, Germany, KB-30BVZ).

## 3. Results and Discussion

**Figure 4:**
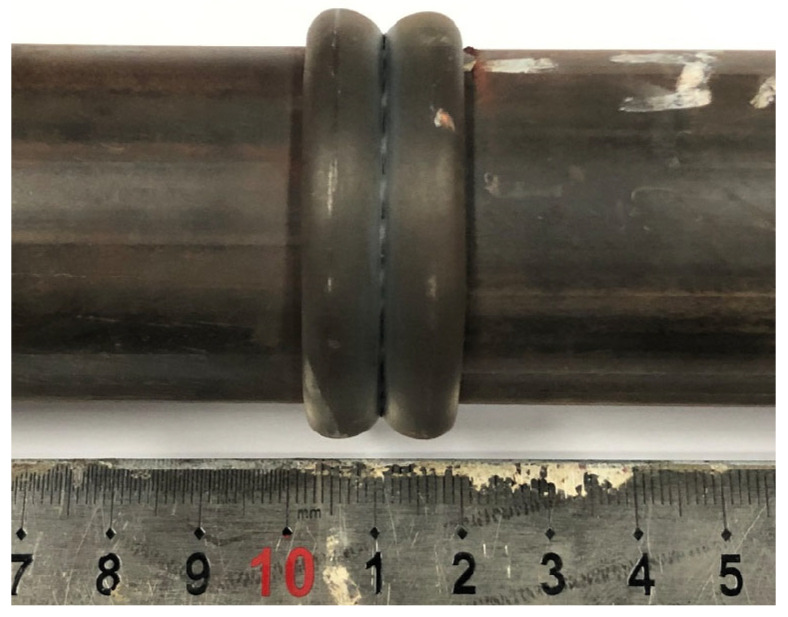
The welded joint.

Figure 4 shows a typical welded joint with a smooth flash that is symmetrically distributed on both sides of the weld and has a width of approximately 17 mm. Unlike other solid-state linear friction welded joints that may exhibit wrinkles on the surface [24,25], the smooth joints obtained in this study indicate that the base metal was uniformly extruded under pressure during the welding process.

### 3.1. Microstructure Evolution

**Figure 5:**
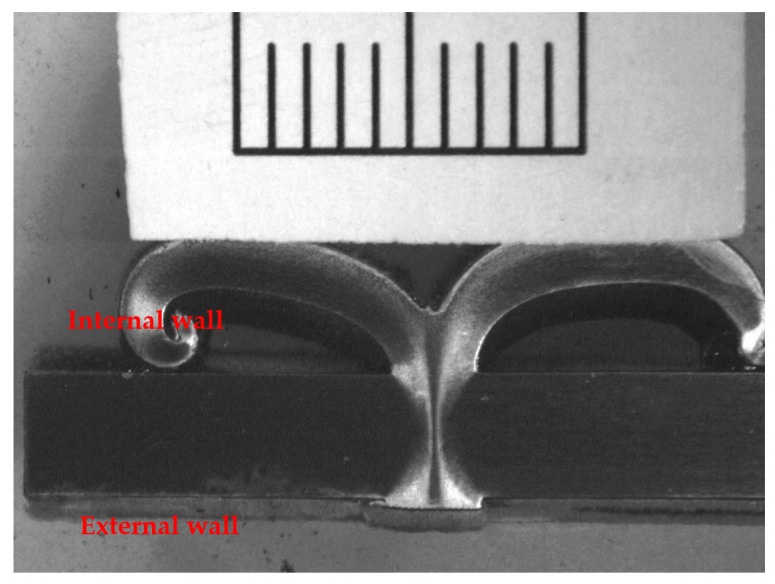
The macroscopic view of the cross-sectional welded joint (the external flash was machined).

Figure 5 provides a macroscopic view of the cross-sectional structure of the welded joint, where the external flash was ground. The welded joint exhibits a symmetrical internal flash that measures 7 mm in length and 3 mm in thickness. The thermomechanical affected zone (TMAZ) has a width of approximately 1 mm at the center of the wall thickness. Moving away from the center, the TMAZ widens. The weld is free of porosity and crack defects.

**Figure 6:**
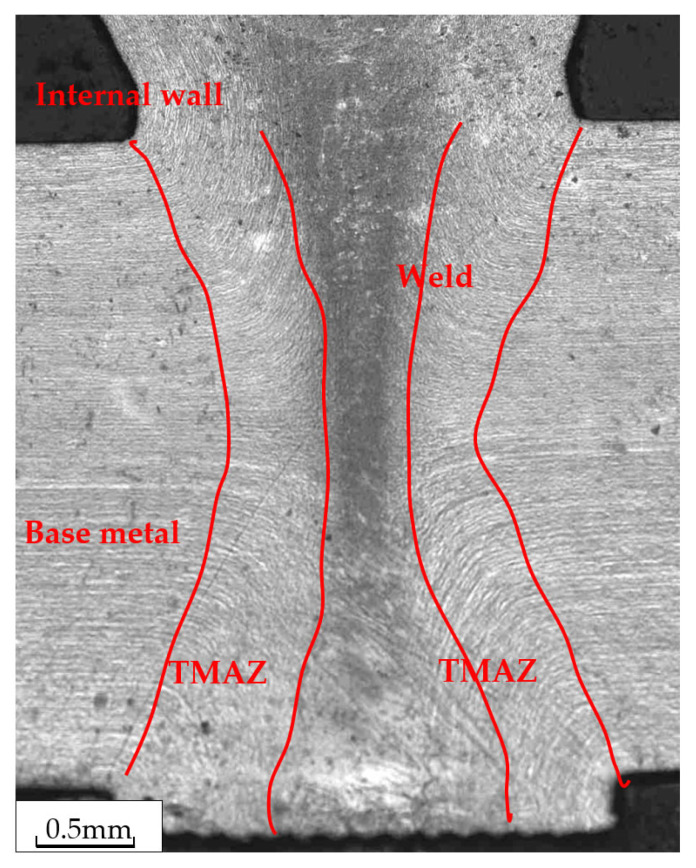
Full view of the microstructure of the welded joint.

Figure 6 illustrates a comprehensive view of the cross-sectional microstructure of the welded joint, which can be divided into three distinct areas: the base metal, the thermomechanical affected zone, and the weld. The ferrite + austenite structure of the base metal is longitudinally distributed along the tube body, forming a strip-like pattern. The thermomechanical affected zone exhibits a streamlined pattern that is consistent with the direction of the flash extrusion. The weld structure appears black in the center of the wall thickness. The microstructure of the flash along the inner side is similar to that of the base pipe, while the microstructure of the flash joint zone is slightly lighter in color, appearing as a light black color.

**Figure 7:**
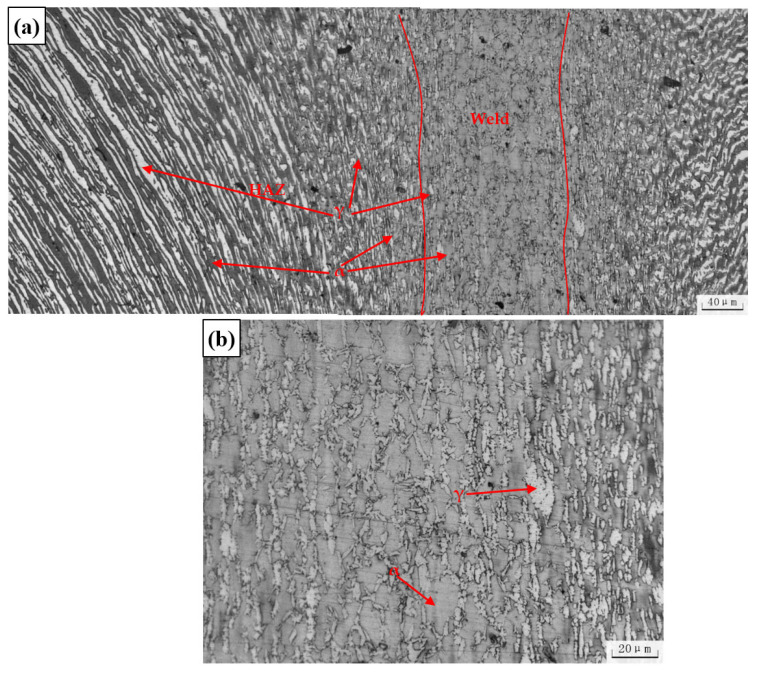
The microstructure of the welded joint. (**a**) The overall microstructure of the welded joint (**b**). High-magnification picture of the weld microstructure.

The metallographic structure of the rotary friction welding joint is depicted in Figure 7. The ferrite + austenite structure of the thermomechanical affected zone (TMAZ) is almost identical to that of the base metal. However, due to the effects of the inertial solid-state welding and frictional heat during the welding process, the austenite strip structure is deflected in the direction of extrusion. In the TMAZ region, the austenite dissolves and becomes spherical and polygonal, a process similar to the dynamic spheroidization of the lamellar α phase in a duplex titanium alloy [26]. In the weld zone, the original austenite is completely dissolved. After the welding process is completed, and the temperature decreases, the austenite precipitates from the ferrite/ferrite boundary to form primary grain boundary austenite.

In comparison to traditional weld joints, the current study does not exhibit the presence of Widmanstätten austenite (WA), intragranular austenite (IGA), or partially transformed austenite (PTA). Instead, grain boundary austenite (GBA) [27] is observed. Typically, during the cooling process of duplex stainless steel welding, austenite is initially precipitated at the ferrite grain boundary due to the relatively high free energy at the boundary. Subsequently, as the cooling progresses, the austenite content at the grain boundary gradually increases, and the nucleation position at the boundary decreases. New crystal nuclei then grow rapidly from the boundary into the ferrite grain in the form of side laths, which are referred to as WA.

In general, the original austenite in hot-rolled pipes undergoes deformation and melting due to frictional heat input and extrusion deformation, resulting in the loss of its distribution in continuous layers. As a result, the amount of austenite in the TMAZ region is reduced. In the weld zone, the original austenite is dissolved, and austenite precipitates at the ferrite grain boundary during the cooling process at temperatures ranging from 1350–800 °C, forming grain boundary austenite. As cooling continues, the nucleation sites of the ferrite grain boundaries decrease, and Widmanstätten austenite forms at the ferrite/austenite grain boundaries, with intragranular austenite forming within the ferrite grains. Due to the relatively rapid cooling rate of solid-phase welding [21,28], which is approximately 10^2^–10^4^ K/s, only grain boundary austenite is found in the weld zone. The absence of σ and chi phases guarantees a good joint quality.

**Figure 8:**
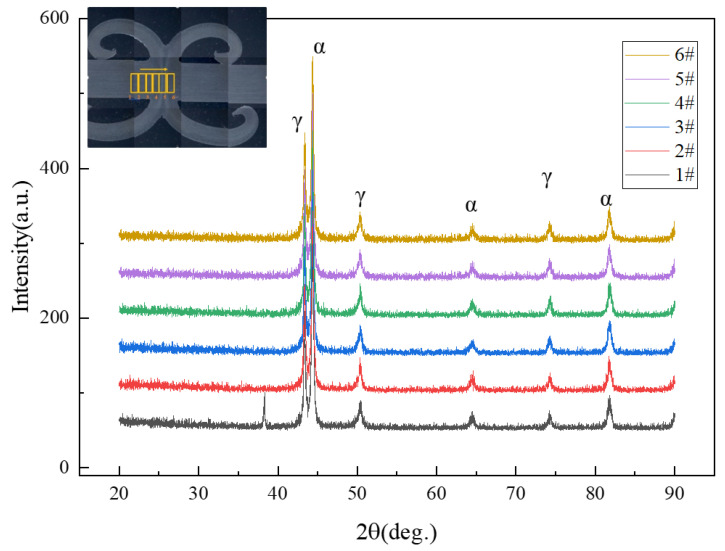
The microstructure of the welded joint.

Figure 8 plots the XRD pattern of the different positions of the welded joint. There are only two phases, ferrite and austenite, without chi and sigma phases, or the volume fraction content is less than the detection limit of XRD (2%) [29]. The results conform to the metallographic analysis.

**Figure 9:**
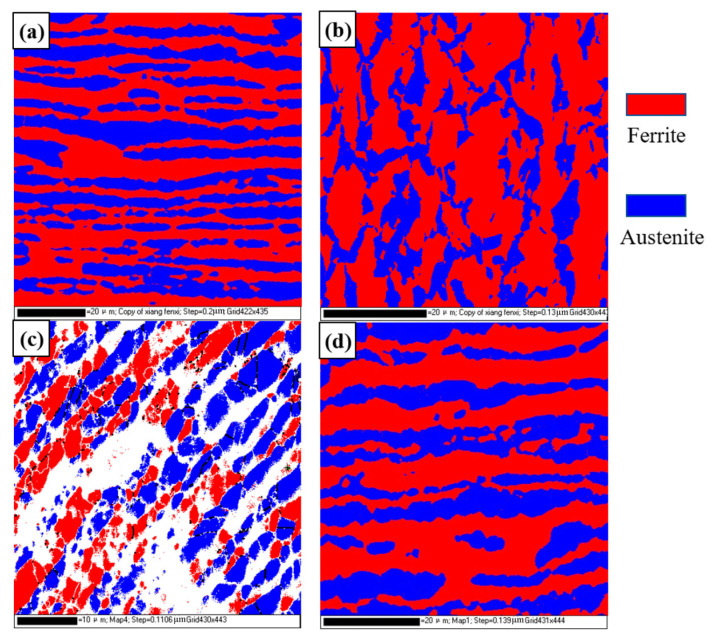
EBSD results of the DSS joint: (**a**) BM; (**b**) TMAZ1; (**c**) TMAZ2; (**d**) weld.

Figure 9 shows the EBSD results of the DSS joint; four regions of interest including the base metal, weld, TMAZ where the microstructure was deformed and extruded (named TMAZ1), and TMAZ at the center of the wall thickness (named TMAZ2) were compared. Due to the large internal stress in the TMAZ1, the EBSD indexing success rate is low, so the result was used for a qualitative comparative analysis. The phase proportion and grain size were obtained from the EBSD data, as shown in Table 2. The proportion of ferrite (53.3%) and austenite (46.7%) in the base metal is close to 1, and the grain sizes are 0.75 μm and 1.16 μm, respectively. The proportion of the TMAZ2 is basically the same as that of the base metal, and the grain sizes are 0.64 μm and 0.47 μm, respectively. In the TMAZ, severe distortion and deformation occurred along the extruded direction, which are due to the large plastic deformation in this zone during the welding process. The phase ratio of this zone is similar to that of the base metal, indicating that the peak temperature in the TMAZ is significantly lower than the α→γ phase transition temperature, and the welding process does not cause a change in the phase ratio, which is similar to the result of friction stir welding [30]. In the weld region, the microstructure and proportion thoroughly changed. It can be inferred that the γ phase in the weld is completely transformed into the α phase. During the cooling process, austenite precipitates from the ferrite phase boundary.

The austenite grains are significantly refined under the thermomechanical effect. In the TMAZ1, the ferrite/austenite grain size is 0.22 μm and 0.24 μm, respectively. In the TMAZ2, the ferrite/austenite grain size is 0.64 μm and 0.47 μm, respectively. The content of ferrite in the weld zone is greater than that of austenite, and the grain sizes are 0.40 μm and 0.41 μm, respectively. The grain size in the TMAZ1/2 and the weld is decreased, and it is refined compared with that of the base metal. Cao et al. [31] used low-temperature friction stir welding to obtain a duplex stainless steel joint with finer grains than those produced by air-cooled friction stir welding, in which the ferrite grain size is 1.11 μm and the austenite grain size is 0.89 μm. Since our base metal grains are relatively fine, the final grain size is even finer.

**Figure 10:**
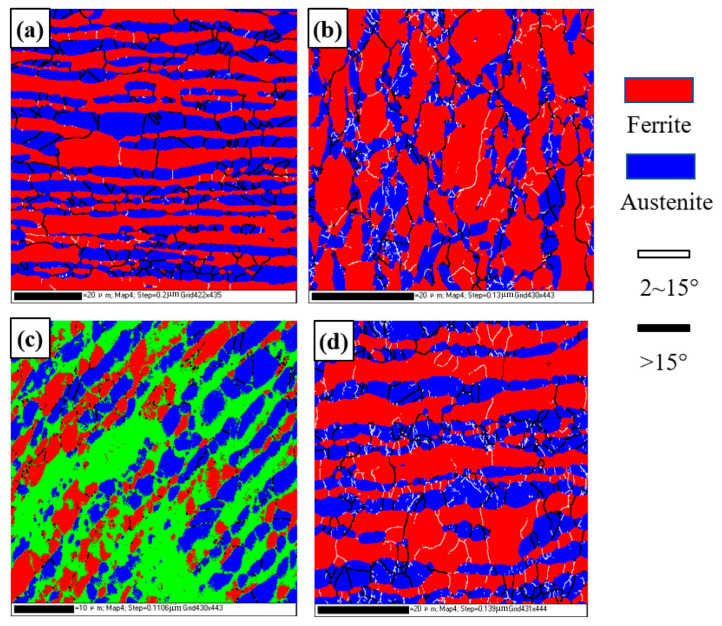

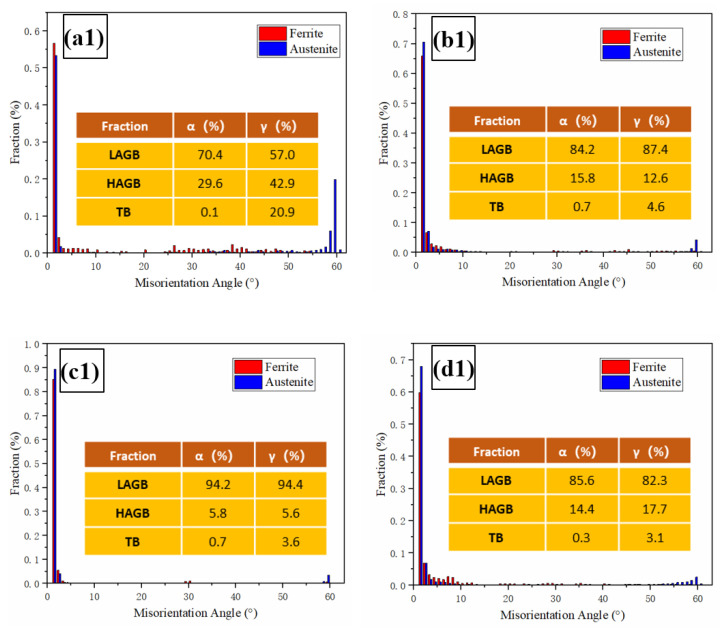
Grain boundary distribution diagram and statistical results for different regions of DSS joints: (**a**) BM; (**b**) TMAZ1; (**c**) TMAZ2; (**d**) weld. (**a1**–**d1**) Misorientation angle statistical results.

Figure 10 shows the grain boundary distribution diagram and statistical results for different regions of the DSS joints. Usually, the grain boundaries, with a misorientation angle that is less than 2°, belong to the EBSD measurement error, and only the grain boundaries with a misorientation angle that is greater than 2° are counted. The 2–15° grain boundary is called the low-angle grain boundary (LAGB), and the grain boundary above 15° is called the high-angle grain boundary (HAGB). The proportion of the small-angle grain boundaries in the base metal is larger than that of the high-angle grain boundaries, which is different from the traditional annealed base metal and is in a metastable state. There are more LAGBs in the weld zone, TMAZ1, and TMAZ2 than the base metal. Generally, dynamic recrystallization (DRX) during the welding process promotes the generation of HAGB, while the generation of LAGB mostly comes from the dynamic recovery (DRV) that occurs within a grain. With the progress of DRX, LAGB gradually develops into HAGB. However, in the process of rotary friction welding, the thermoplastic metal at the welding interface is continuously extruded, and the weld is in a state of dynamic renewal. The thermoplastic metal at the weld is formed under the stable friction stage. In addition, friction welding is a fast-welding process, which has the characteristics of a high heating and cooling rate and short welding time, so there is not enough time for sufficient dynamic recrystallization of the microstructure in the weld zone. Therefore, the LAGB lacks enough driving force and time to develop into the HAGB, so the weld zone mainly shows the characteristics of a sub-grain structure.

**Figure 11:**
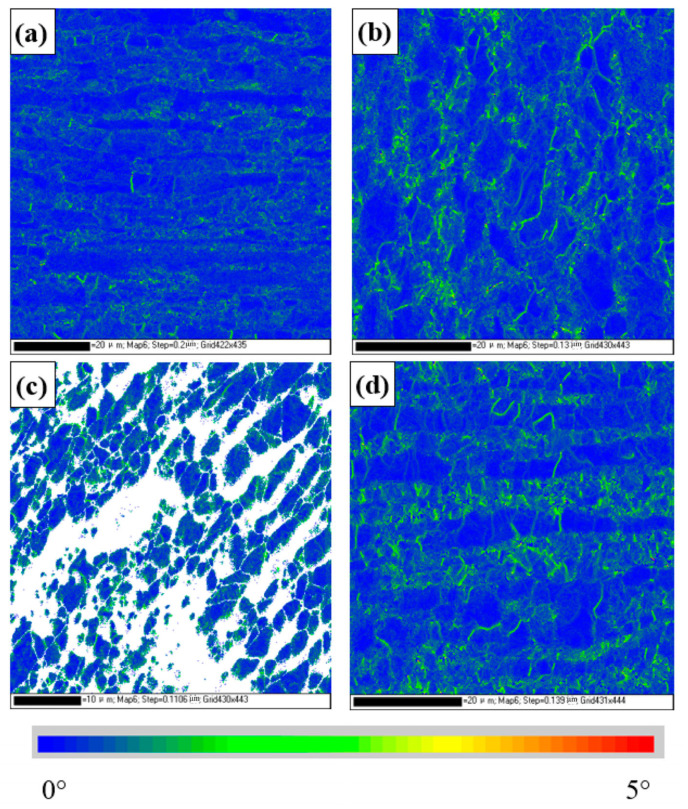

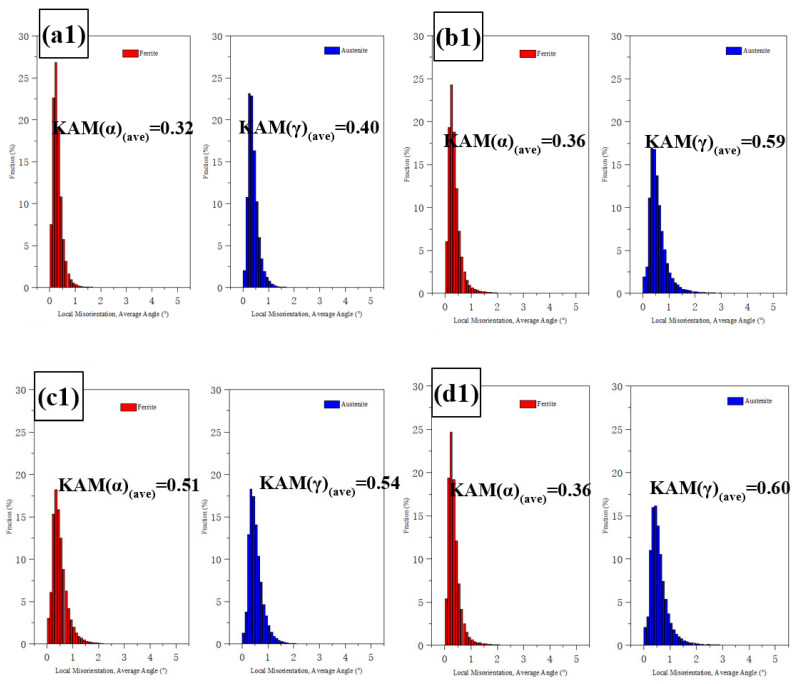
Kernel average misorientation diagram and statistical results for different regions of DSS joints. (**a**) BM (**b**) TMAZ1 (**c**) TMAZ2 (**d**) weld, (**a1**–**d1**) KAM statistical results.

Figure 11 is a kernel average misorientation (KAM) diagram of duplex stainless steel. This KAM map is used to describe the distribution of stored energy and the dislocation density in the local microstructure of the material. In general, KAM is higher in deformed grains, mainly because of the higher dislocation density. In the base metal, the average KAM values of the ferrite and austenite phases are 0.32° and 0.40°, respectively, and the dislocation density of austenite is slightly higher than that of ferrite. In the weld and TMAZ2 regions, due to the extrusion deformation of the friction process, the dislocation density of both ferrite and austenite increased significantly, while the dislocation density of austenite increased more than that of ferrite, which may be due to the lower stacking fault energy.

**Figure 12:**
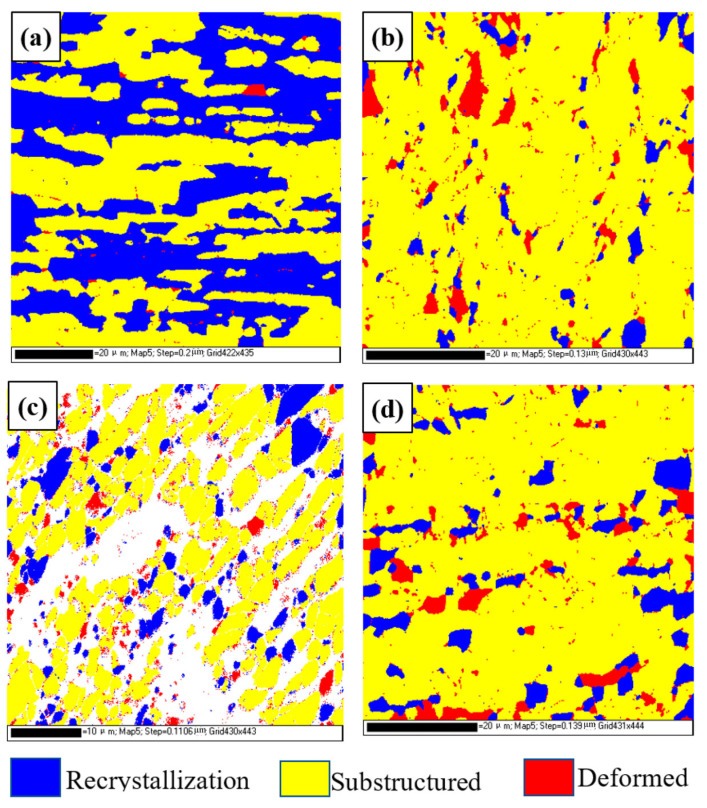
Recrystallization distribution for different regions of DSS joints: (**a**) BM; (**b**) TMAZ1; (**c**) TMAZ2; (**d**) weld.

According to the recrystallization distribution diagram for different regions of the joint shown in Figure 12 and the statistical results shown in Table 3, the recrystallization grains of ferrite in the TMAZ and the weld region are significantly reduced, and the substructure grains are significantly increased. There are the most substructure grains in the weld region, but there is almost no difference in the number of deformed grains. The recrystallized grains of austenite in the weld region decrease, and the substructure grains increase slightly in the TMAZ2, while the weld is basically the same as the base metal. The deformed grains increase significantly in the weld, TMAZ1, and TMAZ2.

**Figure 13:**
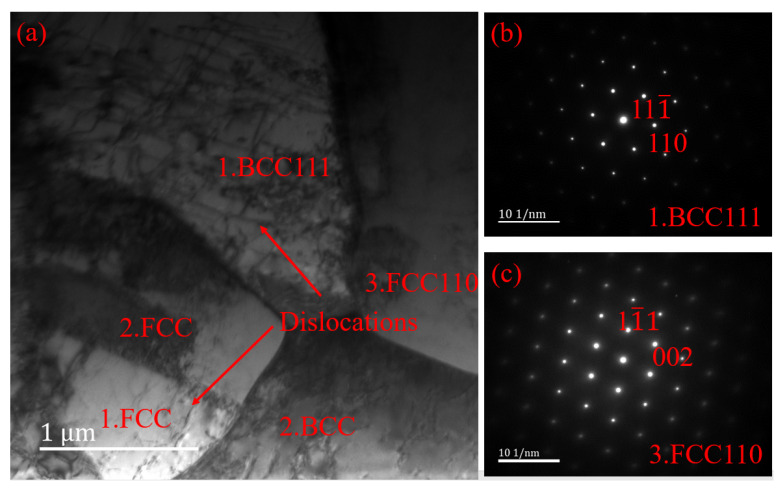
Bright-field image of welded joint: (**a**) bright-field image of welded joint; (**b**) diffraction pattern of ferrite; (**c**) diffraction pattern of austenite.

In order to further characterize the existence of harmful phases in welded joints, since the CrN and sigma phase precipitation are small, phase structure analysis was performed using transmission electron microscopy. Figure 13 shows a bright-field image of the welded joint, and the diffraction of the selected area in the weld zone is in accordance with the ordered body-centered cubic patterns, which are for ferrite, and the ordered face-centered cubic patterns, which are for austenite. There are only austenite and ferrite phases in the weld, and no harmful phases were found in the weld zone. In addition, there are more dislocation densities in the weld in the ferrite and austenite grains, which is consistent with the results observed by EBSD. Compared with friction stir welding joints [31], due to the influence of extrusion deformation and temperature, more dislocations appear in the weld zone.

### 3.2. Mechanical Properties

#### 3.2.1. Tensile Test

**Figure 14:**
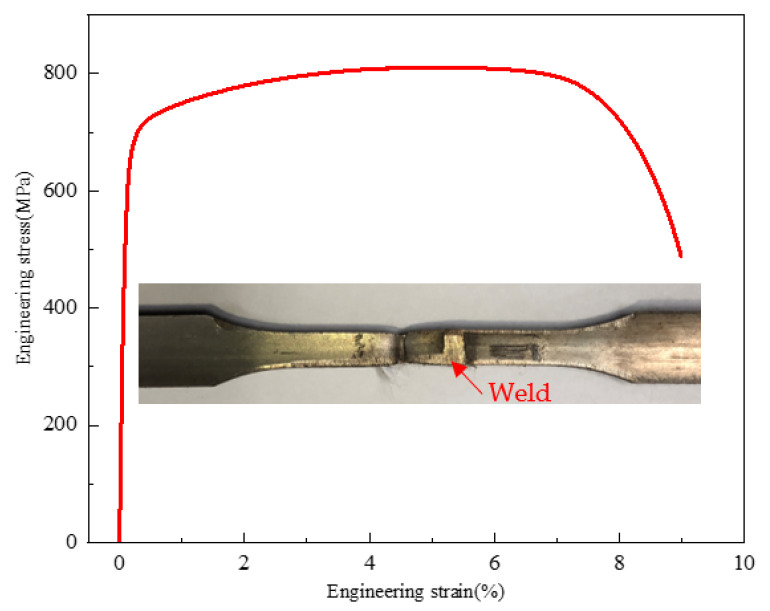
The stress–strain curve of welded joint.

The tensile test curve of the welded joint and the results are shown in Figure 14 and Table 4, respectively. All the welded joints fail at the base pipe, which indicates the good tensile property of the welded joint. The average yield strength of the welded joint is 593.5 MPa, the average tensile strength is 817 MPa, and the two values are almost the same as those of the base material.

The ferrite in duplex stainless steel belongs to the strengthening phase, while the austenite belongs to the ductile phase. Generally, the higher the ferrite content is, the higher the strength and the lower the plasticity are, so the strength of ferritic stainless steel is higher than that of duplex stainless steel, and the strength of duplex stainless steel is higher than that of austenitic stainless steel, though the trend of plasticity is the opposite. Since the ferrite content in the weld zone is higher than that of the base metal, the strength of the weld zone is higher than that of the base metal. The ratio of the two phases of the TMAZ and the base metal is close, so the strengths are similar. During the welding process, the weld and the TMAZ undergo obvious plastic deformation, and this plastic deformation is accompanied by the proliferation of many dislocations, and the entanglement of the proliferated dislocations significantly increases the strength of the weld and the TMAZ. From the results of the KAM and TEM analyses, the dislocations in the weld and the TMAZ proliferate more than those of the base metal, so the strength of the weld and the TMAZ increases.

In addition, according to the Hall–Petch law, as the grain size decreases, the yield strength increases, and, because the average grain sizes of the weld and the TMAZ are greatly refined, the strengths of the weld and TMAZ increase.

#### 3.2.2. Vickers Hardness

**Figure 15:**
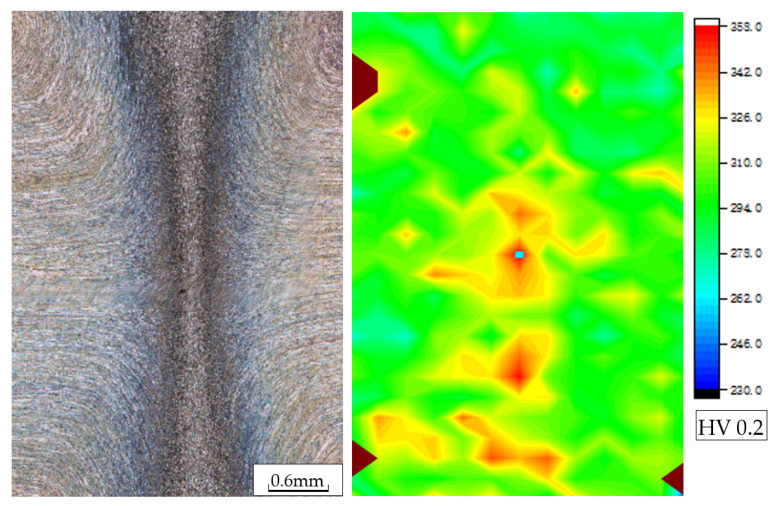
The Vickers hardness (HV0.2) cloud map of the welded joint.

Figure 15 shows the results of the Vickers hardness cloud map of the welded joint. The microhardness of the base metal is about 300HV0.2. In the TMAZ, the microhardness value increases slightly, to about 320HV0.2, and, in the weld region, the hardness value is the highest, at about 358HV0.2. According to G.R. Hitchcock [32], the hardness of ferrite, austenite, and the sigma phase is 344, 290, and 567 kgf/mm^2^, respectively. This is the reason why the strength and hardness of the weld are higher than those of the base metal.

The microhardness of duplex stainless steel is mainly related to the precipitation of secondary phases, the amount and degree of the deformation of the deformed austenite, the ratio of the two phases, and the grain size. When the sigma precipitated phase and Cr_2_N are present, the microhardness increases significantly. The plastic deformation process of austenite promotes the proliferation of many dislocations, and the entanglement of the proliferated dislocations strengthens the austenite and increases the hardness. The finer the grains are, the more obvious the hindrance of the grain boundaries to dislocate is, and the higher the hardness is. Ferrite belongs to the strengthening phase, and the higher the ferrite content is, the higher the hardness is. Therefore, due to a slight increase in the ferrite content in the weld zone, the grain refinement, and an increase in the dislocation density, the hardness in the weld zone increases slightly, and the increase in hardness is not that high due to the absence of precipitated phases.

## 4. Conclusions

In this study, the microstructure and mechanical properties of a rotary friction welded S32205 duplex stainless alloy joint were investigated. The conclusions are drawn as follows:

1. The sound joints, free of cracks and pores, are obtained for the 2205 duplex stainless alloy using rotating friction welding with the following parameters: a rotating speed of 20 m/s, friction pressure of 10 MPa, friction time of 30 s, and forging pressure of 30 MPa.
2. The microstructure of the weld zone is composed of ferrite and austenite phases, with no deleterious phase. The ferrite of the base metal, TMAZ, and weld is 53.3%, 54.5%, and 68.7%, respectively. The weld zone has no harmful phase due to the fast cooling after rotary friction welding.
3. The ferrite/austenite grains are significantly refined under the thermomechanical effect. In the TMAZ1, the ferrite/austenite grain size is 0.22 μm and 0.24 μm, respectively. In the TMAZ2, the ferrite/austenite grain size is 0.64 μm and 0.47 μm, respectively. In the weld, the ferrite/austenite grain size is 0.40 μm and 0.41 μm, respectively. The ultrafine grain DSS weld joint is obtained by rotary friction welding.
4. There are more LAGBs in the weld zone, TMAZ1, and TMAZ2 than the base metal. The generation of LAGB mostly comes from the dynamic recovery (DRV) that occurs within a grain. Due to the high heating and cooling rate and the short welding time of the RFW, there is not enough time for sufficient dynamic recrystallization of the microstructure in the weld zone.
5. Due to a slight increase in the ferrite content in the weld zone, grain refinement, and an increase in dislocation density, the hardness of the weld zone increases to 358 HV0.2 compared with that of the base material, which is about 300 HV0.2, and the strength of the joints is nearly the same as that of the base material.

## Figures and Tables

**Table 1:** Chemical composition (wt. %).

Element	C	Si	Mn	P	S	Cr	Ni	Mo	N
Content	0.018	0.53	1.52	0.025	0.0007	21.57	5.58	2.96	0.17
ISO 13680-2010	≤0.03	≤1.0	≤2.0	≤0.03	≤0.02	21.0~23.0	4.5~6.5	2.50~3.50	0.08~0.20

**Table 2:** The phase proportion and average grain size for different regions of the joint.

	Content (%)		Average Grain Size (μm)	
	Ferrite-bcc	Austenite-fcc	Ferrite-bcc	Austenite-fcc
BM	53.3%	46.7%	0.746	1.156
Weld	68.7%	31.3%	0.404	0.408
TMAZ1	23.3%	32.5%	0.217	0.239
TMAZ2	54.5%	45.5%	0.643	0.471

**Table 3:** Recrystallization distribution results for different regions of DSS joints.

	Ferrite (%)			Austenite (%)		
	Recrystallization	Substructured	Deformed	Recrystallization	Substructured	Deformed
BM	65.9	33.2	0.83	26.2	72.5	1.34
Weld	4.65	94.2	1.17	7.93	73.3	18.8
TMAZ1	26.1	64.8	9.11	10.6	81.2	8.2
TMAZ2	15.7	83.4	0.887	3.29	82	14.7

**Table 4:** Tensile test result.

Sample	Diameter/Width × Thickness × Gauge Length (mm × mm)	Yield Strength (0.5%EUL) (MPa)	Tensile Strength (MPa)	Elongation (%)
Base material	Φ38.1 × 3.2 × 50	603	817	38
		602	819	38
		594	814	38
Welded joint	5 × 3.2 × 25	590	811	Failed at base pipe
		597	823	

## Data Availability

Data available on request due to restrictions e.g., privacy or ethical.
